# Model-Based Patterns of Lymphedema Symptomatology: Phenotypic and Biomarker Characterization

**DOI:** 10.1007/s12609-020-00397-6

**Published:** 2020-11-24

**Authors:** Mei R. Fu, Bradley E Aouizerat, Gary Yu, Yvette Conley, Deborah Axelrod, Amber A. Guth, Jean-Pierre Gagner, Jeanna M Qiu, David Zagzag

**Affiliations:** 1 William F. Connell School of Nursing, Boston College, Chestnut Hill, MA 02467, USA; 2 The Yvonne L. Munn Center for Nursing Research, Massachusetts General Hospital, Boston, MA 02114, USA; 3 Bluestone Center for Clinical Research, Department of Oral and Maxillofacial Surgery, College of Dentistry, New York University, New York, NY, USA; 4 Rory Meyers College of Nursing, New York University, New York, NY, USA; 5 School of Nursing, University of Pittsburgh, Pittsburgh, PA, USA; 6 Department of Surgery, New York University School of Medicine, New York, NY, USA; 7 NYU Perlmutter Cancer Center, New York, NY, USA; 8 Department of Pathology, Microvascular and Molecular Neuro-Oncology Laboratory, NYU Langone Health, New York, NY, USA; 9 Harvard Medical School, Boston, MA, USA; 10 Division of Neuropathology and Department of Neurosurgery, NYU Langone Health, New York, NY, USA

**Keywords:** Symptom, Lymphedema, Phenotype, Biomarkers, Latent class analysis, Limbvolume, Bioimpedance, Symptom distress

## Abstract

**Purpose of the Study:**

More than 50% of breast cancer survivors without a diagnosis of lymphedema suffer daily from numerous and co-occurring lymphedema symptoms. This study aimed to identify lymphedema symptom patterns and the association of such patterns with phenotypic characteristics and biomarkers using latent class analysis (LCA). A prospective, descriptive, and repeated-measure design was used to enroll 140 women and collect data.

**Recent Findings:**

LCA identified three distinct lymphedema symptom classes at 8 weeks and 12 months post-surgery: low, moderate, and severe symptom classes and associated phenotypic characteristics. Participants were more likely to be in the severe symptom classes at 12 months post-surgery if they had lower education level, cording, an axillary syndrome at 8 weeks post-surgery, neoadjuvant chemotherapy, and radiation.

**Summary:**

Pre-surgery level of IL1-a, IL-6, IL-8, and VEGF was associated with the severe symptom class at 8 weeks post-surgery, suggesting that such biomarkers may be used to predict risk for lymphedema symptoms.

## Introduction

Arm lymphedema, an abnormal accumulation of lymph fluid in the ipsilateral upper limb, is a chronic condition for 20–40% of the 3.8 million breast cancer survivors in the USA [1, 2, 3•, 4••, 5]. Attaining the best health possible after breast cancer treatment remains an ongoing challenge since more than 50% of breast cancer survivors without a diagnosis of lymphedema suffer from daily distressing symptoms related to lymphedema (hereafter, lymphedema symptoms) [6••, 7]. Lymphedema symptoms are subjectively perceived indicators of abnormal biological or physiological changes that may or may not be observed objectively [4••, 6••]. Lymphedema symptoms are numerous and typically co-occur, including arm swelling, breast swelling, chest wall swelling, heaviness, firmness, tightness, stiffness, pain, aching, soreness, fibrosis, tenderness, numbness, burning, stabbing, tingling, arm fatigue, arm weakness, and limited movement in shoulder, arm, elbow, and wrist/fingers [6••, 8••]. Such complex symptom experience has been linked to detrimental health outcomes (e.g., disability, psychosocial distress), which are known risk factors for breast cancer survivors’ poor quality of life (QOL) [7•]. Most importantly, lymphedema symptoms may indicate an early stage of lymphedema for which timely intervention may prevent lymphedema from progressing into a chronic condition (i.e., arm lymphedema) that no surgical or medical interventions at present can cure [6••, 9]. Little is known about the patterns of co-occurring lymphedema symptoms (i.e., symptom phenotype) and their associations with the patient characteristics (e.g., demographic, clinical, and behavioral factors), physiological characteristics (i.e., limb volume and lymph fluid level), and patient-reported (i.e., QOL) outcomes.

Besides the unavoidable risk from cancer treatment (e.g., cancer surgery, lymph node procedures, radiation), inflammation and infection are the primary known risk factors for developing lymphedema [10, 11]. Little is known about the association of inflammatory biomarkers with lymphedema symptoms. As part of a larger research project aimed at defining the biological differences that may underlie arm lymphedema and lymphedema symptoms [4••], this study aimed to determine whether latent class analysis (LCA) would aid in the following: (1) the identification of lymphedema symptom patterns and the association of such patterns with demographic, clinical and behavioral factors, physiological outcomes (i.e., increased limb volume, lymph fluid level), and symptom distress; and (2) to evaluate the relationships of inflammatory and lymphatic biology biomarkers with lymphedema symptom patterns over time.

## Background

### Arm Lymphedema

Criteria for defining arm lymphedema remain inconsistent. Research and clinical practice has focused on measuring limb girth, limb volume, limb size, and/or bioimpedance ratio to define the phenotype of arm lymphedema with arbitrarily set criteria of > 2-cm increase in limb girth, > 200-mL limb volume, > 5% increase in limb volume, or estimation of lymph fluid using a bioimpedance ratio ≥ + 10 [3•, 8••]. Such a limited focus on arm lymphedema with inconsistent criteria on its definition has not only stymied precision characterization of the arm lymphedema phenotype(s) but also hampered the evaluation of lymphedema symptom patterns.

Research investigating the biological differences underlying arm lymphedema defined by limb girth, limb volume, limb size, or bioimpedance ratio is limited. To date, the examination of biological differences associated with arm lymphedema after breast cancer has focused primarily on lymphangiogenic and/or inflammatory genetic variations with minimal overlap in the candidate genes assessed [4••, 12, 13•, 14•, 15•]. Of note, lymphedema case definition was different for each cohort and only 16 of the 45 genes were evaluated in more than one cohort with only one of the genes (interleukin 4; *IL4*; which encodes for a multifunctional pro-inflammatory cytokine) associated with an arm lymphedema phenotype in more than one cohort. One study evaluated arm lymphedema defined by bioimpedance ratio and found that an *IL4* polymorphism was associated with arm lymphedema [13•], while another study observed no associations between *IL4* and arm lymphedema phenotype defined by > 5% limb volume increase and lymph fluid level of ≥ + 10 at 12 months following breast cancer surgery, but did observe an association with lymphedema symptoms [4••].

### Lymphedema Symptoms

Multiple lymphedema symptoms usually co-occur, even before swelling can be observed or measured along the trajectory of breast cancer recovery [4••]. While the exact etiology of lymphedema symptoms after breast cancer treatment remains ill-defined, breast cancer survivors are known to have a compromised lymphatic system due to cancer surgery, dissection of lymph nodes and vessels, and radiation, often leading to ineffective lymphatic drainage and accumulated lymph fluid in the affected area or limb. Physiologically, the accumulation of lymph fluid in the affected area or limb can create undue pressure on nerves, producing feelings of pain, aching, tenderness, soreness, burning, tingling, stabbing, and numbness, as well as inducing sensations of swelling, heaviness, tightness, and firmness [8••]. Accumulated lymph fluid in the affected area or limb also leads to stiffness and limited limb movement of the arm, shoulder, elbow, wrist, and fingers. Lymphedema symptoms are associated with accumulation of lymph fluid in the ipsilateral upper limb [8••]. Moreover, the number of symptoms reported is positively correlated with breast cancer survivors’ limb volume and lymph fluid level [6••, 8••].

Defining lymphedema symptom clusters after breast cancer treatment has received limited attention in the field. In addition, research and clinical practice has focused solely on the lymphedema symptom of swelling, likely because of its measurability in terms of limb size, limb volume increase, or lymph fluid level, which has been used to define lymphedema [6••, 8••]. In a previous study, we defined lymphedema symptom clusters by clustering co-occurring lymphedema symptoms employing exploratory factor analysis [4••]. We observed three lymphedema symptom clusters (i.e., fluid accumulation, impaired limb mobility, and pain/discomfort), which may reflect more discrete biological processes than considering limb volume or lymph fluid level alone. Thus, an evaluation of how individuals cluster based on their patterns of lymphedema symptoms represents the next step in empirically defining lymphedema symptom patterns that may differ in their causes and impact on clinical and patient-reported outcomes. In addition, sub-groups of individuals who differ in their experience of lymphedema symptom clusters may in part be explained by biological differences. Defining differences in biomarkers that reflect such biological differences could improve our limited understanding of lymphedema symptomology.

## Methods

### Design and Ethical Consideration

This study utilized a prospective, descriptive, longitudinal, and repeated-measure design to enable phase-specific monitoring of phenotypes and biomarker trajectory prior to surgery (baseline), at 8 weeks and at 12 months post-surgery. This study (IRB # 10–02540) was approved by the Institutional Review Board of a metropolitan cancer center in New York of the USA, and all the participants signed the informed consent.

### Study Participants

The sample of 140 participants was recruited from among consecutively identified, pre-operative patients. Each participant was followed for 12 months after breast cancer surgery in a larger research project [4••]. Study participants were women over age 21 years: (a) newly diagnosed with invasive breast cancer (stage I–III) and scheduled for surgical treatment of lumpectomy or mastectomy, including sentinel lymph node biopsy (SLNB) plus lymph node dissection or axillary lymph node dissection (ALND) and neoadjuvant therapy; (b) willing to provide a blood sample for biomarker data collection; (c) without prior history of lymphedema and breast cancer; and (d) willing to participate in the two research follow-up assessments, that is, at 8 weeks and 12 months post-surgery. Women were excluded if (a) she was diagnosed with breast cancer, but would not undergo surgical treatment as breast surgery and removal of lymph nodes are the major treatment-related risk factors for lymphedema; and (b) she was diagnosed with renal or heart failure, had a cardiac pacemaker or defibrillator, artificial limbs, or pregnant, as the manufacturer suggests that bioimpedance measure may not be accurate under these conditions.

### Procedures

We followed the research procedures used in our prior studies [7•, 8••, 16], including for using the perometry and bioimpedance devices as recommended by the manufacturers, and in the collection of blood samples [3•, 16, 17]. Protection of human subjects was ensured by following the guidelines set forth by the Institutional Review Board. Each participant signed the written study consent.

### Phenotype Measures

#### Lymphedema Symptoms and Symptom Distress

The breast Cancer and Lymphedema Symptom Experience Index (BCLE-SEI) is a valid, reliable, 5-point Likert-type self-report instrument to assess symptoms related to lymphedema or fluid accumulation [6••, 8••, 18, 19]. This instrument consists of two parts, one evaluating the occurrence of lymphedema symptoms and another evaluating QOL in terms of symptom distress. The lymphedema symptom assessment (part 1) assesses impaired limb mobility in shoulder, arm, elbow, wrist, and fingers, arm swelling, breast swelling, chest wall swelling, heaviness, firmness, tightness, stiffness, numbness, tenderness, pain/aching/soreness, stiffness, redness, blistering, burning, stabbing, tingling (pain and needles), hotness, blistering, seroma, limb fatigue, and limb weakness. Symptom distress (part 2) evaluates the adverse impact and suffering evoked by one’s experience of lymphedema symptoms [6••, 8••, 18, 19]. Symptom distress includes dimensions of daily living, social function, sleep disturbance, sexuality, emotional/ psychological distress, days absent from work.

#### Infrared Perometer Measurement

We used the Perometry 350S device to measure each arm. The Perometry generated a 3-dimensional limb image with limb volume calculated for each participant. This optoelectronic method has a standard deviation of 8.9 mL (arm), less than 0.5% of limb volume with repeated measuring [3•, 16, 17].

#### Lymph Fluid Level

We used the Imp XCA® (Impedimed, Brisbane, Australia), a bioelectrical impedance analysis (BIA) device to assess lymph fluid level. The device measures resistance of the extra-cellular fluid in terms of the L-Dex ratio. With the development of lymphedema, the impedance of the limb decreases and the L-Dex ratio increases [16].

#### Demographic, Clinical, and Behavioral Data

Demographic data included age, education, marital status, race, and ethnicity. Clinical data included breast cancer diagnosis, stage of disease, cancer location, surgeries, lymph nodes procedure, neoadjuvant chemotherapy or radiation before cancer surgery, type of adjuvant therapy (radiation or chemotherapy post-surgery or hormonal therapy after cancer surgery), lymphedema diagnosis/treatment, medications, and treatment complications (e.g., infections and cording).

Behavioral information focused on receiving physical therapy for shoulder and arm mobility, weekly physical activity [8••], and engagement of physical activity of vigorous, moderate and light intensity at least 2–3 times a week since their breast cancer surgery [20]. Participants would provide “yes” or “no” answer to each question regarding physical therapy and physical activity.

#### Height and BMI

Height was measured to the nearest 0.1 cm with a portable stadiometer without shoes. An electrical bioimpedance device (InBody 520, Biospace Co., Ltd) was employed to measure weight and the device automatically calculated BMI using the following formula: weight (kg)/height (m^2^).

### Biomarker Data Collection

#### Biomarker Selection

Biomarkers were selected based on previous research on arm lymphedema [4••, 12, 13•, 14•, 15•]. The inflammatory biomarkers evaluated included lymphatic growth factors (vascular endothelial growth factor (VEGF, VEGFC & VEGFD) and cytokines IL1-*a*, IL-4, IL-6, IL-8, IL-10, IL-13, tumor necrosis factor-alpha (TNF-*α*) (Table 1).

#### Blood Sample Collection and Serum Extraction

Biomarker data were obtained from blood specimens collected from each participant at baseline (prior to surgery), and at the 8-week and 12-month post-surgery visits. A phlebotomist drew 3.5 mL of venous blood in Vacutainer SST tubes (BD, Franklin Lakes, NJ, USA) containing a clot activator and serum separator gel between 8:00 AM and 10:00 AM after an overnight fast. After the whole blood in the SST tubes was allowed to clot for 30 min at room temperature, the specimens were transported on ice in a cooler to the designated laboratory where they were centrifuged at 1000g for 15 min to separate the serum and clotted blood. Serum aliquots were stored at − 80 °C until analysis.

#### Serum Biomarker Levels

Serum levels of cytokines were measured using customized V-PLEX human cytokine panel kits from Meso Scale Discovery (MSD, Rockville, MD, USA) according to the manufacturer’s instructions. The method uses multiplex sandwich immunoassays with electrochemiluminescence detection, whereby the cytokines bind to specific capture antibodies immobilized on MULTI-SPOT 96-well microplates and are then labeled with electrochemiluminescent detection antibodies (SULFO-TAG). For this study, serum samples were thawed on ice and assayed in triplicate at a 1:2 dilution in Diluent 7 (MSD). Each microplate measured one or more of the target biomarkers (i.e., human IL-1*α*, IL-4, IL-6, IL-8, IL-10, IL-13, VEGF, VEGFC, VEGFD, and TNF-*α*) together with an 8-point standard curve (0 up to 1000 pg/mL for most cytokines tested or up to 10,000 pg/mL for VEGFC and VEGFD). As controls, samples spiked with 3 different concentrations (low, medium, and high) of recombinant human cytokines (MSD) were assayed. The intensity of the emitted light was measured on MESO QuickPlex SQ120 instrument (MSD) and analyzed using Discovery Workbench 4.0 software (MSD). Sample data read against corresponding linear standard curves covering greater than 4 logs of cytokine concentrations provided a quantitative measure of each serum biomarker level with high sensitivity (lower limit of detection ranging from 0.02 pg/mL for IL-4 to 11.1 pg/mL for VEGFC) and high precision (coefficient of variation ranging from 2.8% for TNF-*α* to 5.8% for IL-4). There were no outliers and missing biomarker data. The levels of the biomarkers of triplicate samples were averaged for data analysis.

### Statistical Analysis

#### Latent Class Analysis

LCA was used to empirically identify classes of individuals reporting similar patterns of lymphedema symptoms [21]. We analyzed the occurrence of 26 lymphedema symptoms and an overall count of symptoms using LCA with varying number of classes, ranging from 1 to 7 [22, 23]. The optimal number of classes was determined using Bayesian information criterion (BIC), which balanced model fit and parsimony [24, 25]. The parameters of the LCA model included the following: (1) the creation of a total symptom count indicator as a simple sum of all symptom items to reflect the cumulative exposure, (2) the probability of each specific symptom being used within each latent class, (3) the overall proportion of the population in each of the latent classes, and (4) the mean number of different symptoms reported in each latent class. The LCA model was fit using maximum likelihood in the Mplus version 6.11 [26], where the dichotomous symptom indicators were modeled with a binomial logit link and the overall count of different symptoms listed was modeled with a log Poisson link. Once the optimal number of classes was determined, the posterior probability that a certain individual belongs to a certain latent class was computed using Bayes’ rule [21]. Qualitative descriptions of the resulting symptom profile classes are based on the prevalence of individual symptoms and types of symptoms and were labeled as severe/low if the prevalence of use within the latent class was above or below the overall sample prevalence by at least 10%.

#### Evaluation of Participant Characteristics and Biomarkers by Lymphedema Symptom Latent Class

We conducted descriptive analyses for demographic and clinical characteristics, physiological outcomes (i.e., limb volume change and lymph fluid level), and biomarkers measured at baseline (pre-surgery), and at 8 weeks and 12 months post-surgery. We compared the participant characteristics and biomarkers across the predicted LCA lymphedema symptom classes using chi-square tests and analysis of variance (ANOVA) for categorical and continuous markers, respectively. We report the median symptom distress subscales (i.e., daily living, social function, emotional/psychological distress, sleep disturbance, sexuality, work outside home, days absent from work) among each class using Kruskal-Wallis tests to examine differences in the median and interquartile ranges of symptom distress subscale scores between classes. Biomarker measurements were mathematically transformed using the natural logarithm (ln) in order to achieve assumptions of normality and the mean ln (biomarker) levels among each class using one-way ANOVA to examine differences in the average and standard deviations of specific biomarker levels between classes. Non-parametric Spearman correlations were used to test for the association between limb volume change, L-Dex ratio at all three time points, and the symptom distress subscale scores at follow-up. Statistically significant differences among the three groups were further evaluated by post hoc comparison of sub-groups using a Bonferroni correction for the three pairwise tests performed (*p* = 0.05/3 = 0.017). All point estimates were generated with 95% confidence intervals at which significance level of less than 0.05. STATA (version 14) was used for all analyses.

## Findings

Of the 140 patients enrolled in the study, a total of 136 participants completed the study (2.9% attrition rate). The participants were women with a mean age of 52 years (range from age 26 to 81 years) and at least a bachelors’ degree (66.9%), being married (58.8%), and employed (83.1%). The participants were diverse in both racial and ethnic background: 60.3% were white, followed by Black/African American (19.9%), Asian (9.6%), and Hispanic (8.8%). The majority of the participants received adjuvant chemotherapy (70.7%) and radiation (70.1%). Among all the participants, 48.5% had lumpectomy and 51.5% mastectomy.

### Latent Class Analysis Model-Based Patterns of Lymphedema Symptomatology

Prior to surgery, only one patient reported having 8 lymphedema symptoms and 18 had 1–7 symptoms while the rest of the patients reported no lymphedema symptoms; all of these participants who reported symptoms at baseline had received neoadjuvant chemotherapy or radiation prior to surgery. At 8 weeks post-surgery, 82% of participants reported at least 4 lymphedema symptoms. No patients reported symptoms of hand swelling or blistering. The best fitting LCA model of lymphedema symptoms was a three-class solution: (1) a low symptom class (mean 4.2 symptoms) with less than average prevalence of all reported symptoms; (2) a moderate symptom class (mean 8.8 symptoms) defined by a higher than average prevalence of limited shoulder movement (93%), limited arm movement (96%), and arm tightness (83%); and (3) a severe symptom class (mean 14.8 symptoms) with a higher than average prevalence of 21 symptoms. Of patients in the severe symptom class, 100% of them reported tenderness (100%) and chest wall swelling (100%). Over 80% of patients in the severe symptom class reported breast swelling (96%), arm tightness (83%), arm stiffness (88%), and numbness (88%). It should be noted that 8% of patients in the severe symptom class reported symptoms of limited wrist and elbow movement while no patients reported such symptoms in the low and moderate symptom class. The LCA also estimated the proportion of participants in each class: the low symptom class was the largest (48%), followed by the moderate symptom class (34%) and a severe symptom class (18%). The overall prevalence of different symptoms reported and the results of the prevalence within the classes identified by the LCA are shown in Table 2. Overall, participants reported an average of 7.6 symptoms, 85% reported tenderness, 59% reported breast swelling, and 62% reported pain/aching/soreness at 8 weeks post-surgery.

At 12 months post-surgery, the LCA model also identified three distinct lymphedema symptom classes: (1) a low symptom class (mean 1.2 symptoms) with less than average prevalence of all reported symptoms; (2) a moderate symptom class (mean 5.9 symptoms); and (3) a severe symptom class (mean 14.0 symptoms) defined by a higher than average prevalence of all 26 symptoms. It should be noted while no patients reported hand swelling and blistering at 8 weeks post-surgery, 57% of patients in the severe symptom class at 12 months post-surgery reported hand swelling, and 9% reported blistering. In addition, no patients in the low and moderate symptom class at 12 months post-surgery reported blistering. Table 3 presents the overall prevalence of different symptoms reported and the results of the prevalence within the classes identified by the LCA at 12 months post-surgery. The moderate symptom class was the largest (46%), followed by the low symptom class (37%) and the severe symptom class (17%). There was a significant association between the latent classes at 8 weeks and at 12 months (*X*^2^ = 11.4; df = 4; *p* = 0.023).

### Patient Characteristics Associated with the Lymphedema Symptom Classes

#### Demographic, Clinical, and Behavioral Factors

Participants who were older, higher weight and BMI, and had more lymph nodes removed were more likely to be in the severe symptom class at 8 weeks post-surgery, compared to those in the moderate and lower symptom classes. At 8 weeks post-surgery, breast reconstruction and mastectomy were associated with being in the moderate and severe symptom class, while lumpectomy was associated with being in the lower symptom class (*p* = 0.002). Participants in the severe symptom classes at 8 weeks and 12 months post-surgery reported more episodes of infection during the time of study (*p* = 0.018 and *p* = 0.001, respectively). Patients who reported to engage in moderate physical activity 2–3 times per week were more likely to be in the moderate symptom class at 8 weeks post-surgery (*p* = 0.007) (Table 4). Participants reporting cording, an axillary syndrome, at 8 weeks post-surgery were more likely to be in the severe symptom classes at 8 weeks and at 12 months post-surgery (*p* < 0.001 and *p* = 0.015, respectively). Participants in the moderate or severe symptom classes at 12 months post-surgery were more likely to have lower education level with an associate degree or less (*p* = 0.016) and were more likely to have neoadjuvant chemotherapy (*p* = 0.021) and radiation (*p* = 0.001) (Table 5).

#### Physiological Outcomes of Limb Volume and Lymph Fluid

The moderate and severe lymphedema symptom classes were associated with greater lymph volume change and larger bioimpedance ratio. Participants in the severe symptom class were more likely to have a higher median lymph volume change at 12 months post-surgery (6 vs 1 vs − 1%; *p* = 1.1) than those in the moderate and low symptom classes. In terms of lymph fluid levels, participants in the severe symptom class were more likely to have a higher median L-Dex level at 8 weeks (4.7 vs 1.15 vs − 0.4; *p* = 0.002) and 12 months post-surgery (4.4 vs 0.15 vs − 1.0; *p* < 0.001) than their counterparts in the moderate and low symptom classes. The L-Dex ratio at 12 months post-surgery was also able to detect a pairwise difference in the moderate vs severe symptom class (4.40 vs 0.15; *p* < 0.001). In addition, the lower symptom class can be differentiated from the severe symptom class in terms of the median L-Dex both at 8 weeks (4.7 vs − 0.4) and 12 months post-surgery (4.4 vs − 1.0) (Table 6), while lymph volume change differed only at 12 months post-surgery (6 vs − 1%) (Table 7).

#### QOL in Terms of Symptom Distress

Significant differences were found among the three lymphedema symptom classes at 12 months post-surgery (*p* = 0.0001) in terms of symptom distress subscales of impaired daily living, social distress, emotional distress, impaired self-perception, sleep disturbance, impaired sexuality, work outside home, and days absent from work (Table 8). Higher median values of all eight symptom distress subscales were observed in the severe symptom class, suggesting higher distress from lymphedema symptoms. In addition, significant symptom distress was also found in the moderate symptom class in comparison to the low symptom class in terms of the following symptom distress subscales: impaired daily living, emotional distress, impaired self-perception, sleep disturbance, work outside home, and days absent from work (all post hoc Bonferroni-corrected *p* < 0.05) (Table 8).

#### Inflammatory Biomarkers in Relation to Lymphedema Symptom Classes

Levels of IL1-*α* pre-surgery (*p* = 0.0012) and at 8 weeks post-surgery (*p* = 0.0079) were significantly lower in the severe as compared to the low symptom class at 8 weeks post-surgery, IL-8 levels at baseline were significantly lower in the severe as compared to the low symptom class (*p* = 0.0334) at 8 weeks post-surgery, and IL-6 levels at baseline were significant higher in the severe as compared to the low symptom class at 8 weeks post-surgery (*p* = 0.0168) (Table 9). Levels of VEGF at 8 weeks post-surgery were significantly higher in the severe as compared to the low symptom class at 8 weeks post-surgery (*p* = 0.0029). Pre-surgery levels of VEGF showed a trend towards higher levels in the more severe as compared to the low symptom class at 8 weeks post-surgery (*p* = 0.0681). Pre-surgery levels of VEGF were significantly elevated in the severe as compared to the low symptom class at 12 months post-surgery (*p* = 0.0477) (Table 10). Levels of VEGF at 8 weeks post-surgery showed a trend towards higher levels in the more severe as compared to the low symptom class at 12 months post-surgery (*p* = 0.0029). Pre-surgery levels of IL-4 showed a trend towards lower levels in the severe as compared to the low symptom class at 8 weeks post-surgery (*p* = 0.0525). Levels of IL-4 at 8 weeks post-surgery also showed a trend towards lower levels in the more severe as compared to the low symptom class at 8 weeks post-surgery (*p* = 0.0747). Levels of IL-6 at 8 weeks post-surgery showed a trend towards lower levels in the more severe as compared to the low symptom class at 8 weeks post-surgery (*p* = 0.0835). Pre-surgery levels of IL-10 showed a trend towards lower levels in the more severe as compared to the low symptom class at 8 weeks post-surgery (*p* = 0.0835). Baseline levels of VEGFC (*p* = 0.0938) and VEGFD (*p* = 0.0571) showed trends towards lower levels in the more severe as compared to the low symptom class at 12 months post-surgery. The median and IQR for each biomarker in terms of symptom classes at 8 weeks and 12 months are presented in Tables 11 and 12.

## Discussion

Very few research studies have focused on lymphedema symptomology. The current study is the first to define lymphedema symptom patterns using LCA to empirically identify clusters of individuals reporting similar patterns and trajectories of lymphedema symptoms. The LCA model identified low, moderate, and severe symptom classes at both 8 weeks and 12 months post-surgery and there was a significant association between the latent classes at 8 weeks and at 12 months. About 18% of patients were classified into the severe symptom classes at 8 weeks and 12 months post-surgery, which consisted of about 14 symptoms and characterized by a higher than average prevalence of almost all 26 symptoms. Women in the severe symptom class had the highest lymph fluid level and limb volume and that could be classified as arm lymphedema based on current criteria [3•, 16]. The moderate symptom classes also had higher lymph fluid level and limb volume change in comparison with the low symptom classes, a finding which may serve as initial evidence that patients in the moderate symptom classes are at-risk patients. Given the incurable and progressive nature of arm lymphedema and the fact that early intervention enables better clinical outcome, it is extremely important to provide timely intervention to patients in the moderate symptom classes.

A major goal of lymphedema symptom science is to reduce symptom distress and improve QOL among breast cancer survivors. The low symptom classes were characterized by the lowest reported symptom distress. Severe symptom distress was observed in the 12-month post-surgery severe symptom class in all symptom subscales: impaired daily living, social distress, emotional distress, impaired self-perception, sleep disturbance, impaired sexuality, work outside home, and days absent from work in the past month. The severity of symptom distress in the severe symptom class makes it imperative to provide effective intervention to improve their symptom experience and QOL. It is also important to note that significant symptom distress was found in the moderate symptom class in comparison to the low symptom class in all the symptom distress subscales except social distress and impaired sexuality: impaired daily living, emotional distress, impaired self-perception, sleep disturbance, work outside home, and days absent from work. Our findings provide initial evidence that breast cancer survivors in the moderate symptom class should also receive an intervention(s) to improve their symptom experience and decrease symptom distress or even reduce their risk for progressing to the severe symptom class.

Precision characterization of lymphedema symptom patterns or clusters is essential to laying the foundation for defining the underlying mechanisms that may lead to a cure. Our study demonstrated that the severe and moderate symptom classes shared the same demographic and clinical characteristics of arm lymphedema phenotype: lower level of education, receiving neoadjuvant chemotherapy and radiation, and having breast reconstruction [2, 11]. Inflammation/infection has been identified as an important predictor for arm lymphedema [10]. This prospective study found that patients in the severe symptom class at 8 weeks post-surgery reported more episodes of infection during the time of study and more episodes of infection at 12 months post-surgery. Perhaps, intense intervention at 8 weeks post-surgery for women in the severe symptom class may help to prevent infection during the first year of surgery and in turn reduce the risk of lymphedema.

Cording or axillary web syndrome is one of the common post-surgical complications among breast cancer survivors. Our study found that patients with cording were more likely to be in the severe or moderate symptom classes. Although the biological mechanism underlying cording is ill-defined, inflammation is assumed to be the major cause. Physical activity is important to help lymph fluid flow as patients in the study who reported to engage in moderate physical activity 2–3 times per week were more likely to be in the moderate symptom class at 8 weeks post-surgery. More research needs to be done to further elucidate the effect of physical activity on lymphedema symptom classes. Identification of characteristics such as inflammation/infection and physical activity provide a foundation for future investigation on how these characteristics may influence symptom class membership and be related to differences in biomarkers (e.g., serum/plasma protein, gene expression, epigenetic regulation).

A long-term goal of our research is to advance symptom science by achieving precision assessment and early detection of lymphedema symptom classes and defining the environmental and patient (e.g., demographic, clinical, biomarker) differences that distinguish lymphedema symptom classes in order to devise approaches to decrease symptom distress and improve QOL among breast cancer survivors. Our current study identified significant differences in biomarkers (i.e., IL1-*α*, IL-8, IL-6, VEGF) prior to surgery in the moderate and severe symptom classes. Changes in these inflammatory mediators prior to surgery were also associated with class membership post-surgery. The association between symptom classes and biomarker levels attenuated at 8 weeks and 12 months post-surgery. A striking finding is that differences in biomarkers at baseline (prior to surgery) were associated with latent class membership at 8 weeks and 12 months post-surgery. This finding is significant because it suggests that biomarkers may be used to predict subsequent risk for lymphedema symptoms and potentially even arm lymphedema after surgery. Levels of IL1-*α*, IL6, IL8, and VEGF at baseline were associated with latent class membership at 8 weeks post-surgery.

Inflammation and infection are established risk factors for risk of arm lymphedema [10, 11]. IL1-*α* is an acutephase reactant that promotes inflammation and is expressed in lymph node, epithelial cells, and fibroblasts [27]. Serum levels of IL1-*α* in healthy persons are estimated to occur in concentrations of less than 1 pg/mL [28]. Elevated levels of IL1-*α* at baseline for individuals in the low symptom class as compared to the moderate and severe symptom classes suggest that the IL1-*α*-mediated pro-inflammatory response may be more robust in patients who are in the low symptom class which could lead to a more efficient response to infection (i.e., more rapid resolution of inflammation due to infection). This pattern of IL1-*α* was also observed in serum levels at 8 weeks post-surgery in relation to latent class membership at 8 weeks post-surgery.

Serum IL-6 in healthy persons ranges from 1 to 10 pg/mL [29]. IL-6 is lower than average in the low and moderate as compared to severe symptom class. IL-6 has both pro- and anti-inflammatory functions and is produced by a number of cell types including immune cells, adipocytes, and myocytes. The lack of an increase in TNF-*α* in the context of increased levels of IL-6 in the severe symptom class suggests that IL-6 may be acting primarily as an anti-inflammatory mediator, in part by its suppression of TNF-*α* and IL-1. Taken together, we speculate that these effects could culminate in impaired response to infection resulting in prolonged infection and damage to the lymphatic system, resulting in more severe lymphedema symptoms and risk for arm lymphedema.

Serum IL-8 in healthy persons ranges from 1 to 10 pg/mL [29]. IL-8 is lower in low and moderate as compared to severe symptom class at 8 weeks and also at 12 months post-surgery. IL-8 is a potent chemoattractant that functions early in the innate immune response. Congruent to the observed findings for IL1-*α* and IL-6, we speculate that lower IL-8 levels in individuals who are in the severe symptom class may have a less robust inflammatory response which may result in prolonged infection and damage to the lymphatic system, resulting in more severe lymphedema symptoms and risk for arm lymphedema.

The observation that VEGF levels at baseline and 8 weeks post-surgery were increased in the severe symptom class as compared to the low and moderate classes at both 8 weeks and 12 months post-surgery is intriguing. VEGF is associated with cancer survivorship and cancer recurrence [30]. As lymphedema is a disease of the lymphatic and immune systems, further longitudinal exploration of the impact of severe lymphedema symptoms is needed on cancer recurrence and survival. Further exploration of these biomarkers, including their regulation and downstream targets, is critical to improve our understanding of the biological differences that characterize lymphedema symptom clusters, which is essential for finding either effective interventions for lymphedema symptoms or even a cure for lymphedema. Future studies are warranted to replicate our observation that differences in IL1-*α*, IL-6, IL-8, and VEGF levels and lymphedema symptoms and to evaluate levels of these biomarkers in relation to lymphedema symptom classes and arm lymphedema in an independent sample.

## Limitations and Strength of the Study

Although our sample size was adequate for an exploratory study, we are aware of the limitations of sample size, limited scope of biomarkers evaluated, and having only 12 months of follow-up. The strengths of our study include a well-designed conceptual model, a prospective approach, and consecutive repeated measurements of phenotypes and biomarkers that enable the observation of changes in phenotypes and biomarkers at meaningful time points. The use of a valid and reliable instrument to evaluate lymphedema symptoms enables a more rigorous assessment of lymphedema symptom phenotypes and to assess symptom distress specifically evoked by lymphedema symptoms. Selection of biomarkers based on well-established risk factors of inflammation and infection is also the strength of our study.

## Conclusion

This prospective study has demonstrated strong evidence that multiple lymphedema symptoms occur concurrently following breast cancer surgery. LCA of the occurrence of multiple lymphedema symptoms is able to detect three distinct symptom class profiles at 8 weeks and 12 months post-surgery. The association of inflammatory biomarkers at baseline with symptom classes at 8 weeks post-surgery suggests that such biomarkers may be used to predict risk for lymphedema symptoms and even arm lymphedema. The observation that the severe symptom class exhibiting the highest lymph fluid level and limb volume, followed closely by the moderate symptom class, suggests that the moderate symptom class may indicate an early stage of arm lymphedema. It should be noted that the detection of significant symptom distress between low and moderate symptom classes is not observed using the current, arbitrary criteria of objective measures of limb volume or lymph fluid level, but is with the LCA model-based symptom classes. This indicates that symptom class may be more sensitive to identify women at risk for the development of arm lymphedema. Timely interventions should be provided to patients in this early prodromal stage to prevent arm lymphedema from progressing into a chronic condition.

## Figures and Tables

**Table 1:** Selected biomarkers and functions

Biomarker	Function
IL1-*α*	Pro-inflammatory; primary initiator of inflammatory response
IL-4	Inflammation and wound repair
IL-6	Pro-inflammatory, primary initiator of inflammatory response, regulating adipose accumulation; also displays anti-inflammatory properties
IL-8	Pro-inflammatory, activation of neutrophils
IL-10	Anti-inflammatory, regulates T cell and macrophage function
IL-13	Anti-inflammatory, inhibits the production of pro-inflammatory cytokines and chemokines, including IL-1*α*, IL-6, & IL-8
VEGF	Vascular permeability factor, creation of new blood vessels after injury, new blood vessels to bypass blocked vessels, muscles after exercises.
VEGFC	Lymphatic-specific growth factor, strong regulator of lymphangiogenesis, hyperplastic lymphatic vessels, lymphedema. VEGFC binds to and activates VEGFR3 and VEFGR2 receptors on lymphatic epithelium.
VEGFD	Lymphatic-specific growth factor receptor, related to lymphedema
TNF-*α*	Pro-inflammatory, key immune mediator

**Table 2:** Latent class analysis model. Prevalence and lymphedema symptom counts within each class at 8 weeks post-surgery

Symptoms	Average symptom class *n* = 136 (100%)	Low symptom class *n* = 66 (48%)	Moderate symptom class *n* = 46 (34%)	Severe symptom class *n* = 24 (18%)
Limited shoulder movement	70 (51%)	7 (11%)	*43 (93%)*	20 (83%)
Limited elbow movement	16(12%)	0 (0%)	9 (20%)	*7 (29%)*
Limited arm movement	67 (49%)	2 (3%)	*44 (96%)*	20 (83%)
Limited wrist movement	1 (1%)	0 (0%)	0 (0%)	2 (8%)
Limited fingers movement	1 (1%)	0 (0%)	0 (0%)	2 (8%)
Arm swelling	15 (11%)	2 (3%)	4 (9%)	*9 (38%)*
Breast swelling	80 (59%)	36 (55%)	21 (46%)	*23 (96%)*
Chest wall swelling	72 (53%)	23 (35%)	25 (54%)	*24 (100%)*
Arm tightness	75 (55%)	17 (26%)	*38 (83%)*	*20 (83%)*
Arm heaviness	53 (39%)	10 (15%)	25 (54%)	*18 (75%)*
Arm firmness	29(21%)	6 (9%)	11 (24%)	*12 (50%)*
Arm stiffness	61 (45%)	6 (9%)	34 (74%)	*21 (88%)*
Pain/aching/soreness	84 (62%)	33 (50%)	32 (70%)	*19 (79%)*
Tenderness	116 (85%)	48 (73%)	44 (96%)	*24 (100%)*
Stabbing	19(21%)	11 (17%)	1 (2%)	*16(67%)*
Tingling	48 (35%)	20 (30%)	11 (24%)	*17(71%)*
Numbness	73 (54%)	31 (47%)	22 (48%)	*21 (88%)*
Burning	33 (24%)	11 (17%)	5 (11%)	*16(67%)*
Arm fatigue	38 (28%)	6 (9%)	17 (37%)	*15 (63%)*
Arm weakness	37 (27%)	4 (6%)	16(35%)	*17(71%)*
Fibrosis (toughness or thickness of skin)	5 (4%)	1 (2%)	0 (0%)	*4 (17%)*
Arm hotness	14 (10%)	4 (6%)	1 (2%)	*8 (33%)*
Arm redness	16(12%)	3 (5%)	1 (2%)	*12 (50%)*
Seroma (pocket of fluid developed)	34 (25%)	22 (33%)	0 (0%)	*10 (40%)*
Hand swelling	0 (0%)	0 (0%)	0 (0%)	0 (0%)
Blistering	0 (0%)	0 (0%)	0 (0%)	0 (0%)
Symptom count	7.584	4.150	8.795	14.791

Italicized values indicate the latent class that exhibits the greatest prevalence of a given symptom

**Table 3:** Latent class analysis model. Prevalence and lymphedema symptom counts within each class at 12 months post-surgery

Symptoms	Average symptom class *n* = 136 (100%)	Low symptom class *n* = 51 (37%)	Moderate symptom class *n* = 62 (46%)	Severe symptom class *n* = 23 (17%)
Limited shoulder movement	41 (30%)	3 (6%)	24 (38%)	*14 (60%)*
Limited elbow movement	8 (6%)	0 (0%)	4 (6%)	*4 (17%)*
Limited arm movement	38 (28%)	0 (0%)	21 (34%)	*17 (74%)*
Limited wrist movement	10 (7%)	1 (2%)	1 (2%)	*8 (35%)*
Limited fingers	10 (7%)	1 (2%)	2 (3%)	*7 (30%)*
Arm swelling	31 (23%)	1 (2%)	12 (19%)	*18 (78%)*
Breast swelling	41 (30%)	6 (12%)	24 (39%)	*11 (48%)*
Chest wall swelling	22 (16%)	1 (2%)	11 (18%)	*10 (43%)*
Arm tightness	60 (44%)	7 (14%)	36 (58%)	*17 (74%)*
Arm heaviness	46 (34%)	0 (0%)	28 (45%)	*18 (78%)*
Arm firmness	22 (16%)	0 (0%)	11 (18%)	*11 (48%)*
Arm stiffness	48 (35%)	2 (4%)	27 (44%)	*18 (78%)*
Pain, aching, or soreness	60 (44%)	6 (12%)	32 (52%)	*22 (96%)*
Tenderness	52 (38%)	1 (2%)	30 (48%)	*21 (91%)*
Stabbing	14 (10%)	0 (0%)	4 (6%)	*9 (39%)*
Tingling	52 (38%)	14 (28%)	22 (36%)	*15 (66%)*
Numbness	58 (43%)	15 (29%)	27 (44%)	*17 (74%)*
Burning	14 (10%)	1 (2%)	4 (6%)	*8 (35%)*
Arm fatigue	30 (22%)	0 (0%)	17 (27%)	*13 (57%)*
Arm weakness	39 (29%)	2 (4%)	20 (32%)	*18 (78%)*
Fibrosis (toughness or thickness of skin)	14 (10%)	1 (2%)	3 (5%)	*10 (43%)*
Arm hotness	11 (8%)	0 (0%)	0 (0%)	*11 (48%)*
Arm redness	5 (4%)	0 (0%)	2 (3%)	*4 (17%)*
Seroma (pocket of fluid developed)	14 (10%)	0 (0%)	5 (8%)	*9 (39%)*
Hand swelling	22 (16%)	3 (6%)	6 (10%)	*13 (57%)*
Blister	1 (1%)	0 (0%)	0 (0%)	2 (9%)
Symptom count	5.460	1.162	5.948	14.013

Italicized values the latent class that exhibits the greatest prevalence of a given symptom

**Table 4:** Demographic and clinical characteristics by symptom risk classes at 8 weeks post-surgery

	Low symptom class	Moderate symptom class	Severe symptom class	*p* value
	Mean (SD)	Mean (SD)	Mean (SD)	
Age	53.32 (10.33)	48.89 (10.76)	55.13 (12.73)	0.0394 ^a^
Weight (pounds)	161.43 (36.27)	147.52 (27.70) B	176.70 (40.33) B	0.0035 ^a^
BMI	28.47 (6.47) A	25.08 (4.71) AB	30.82 (6.89) B	0.0005 ^a^
Education	*n* (%)	*n* (%)	*n* (%)	0.199^b^
Associates degree or less	23 (34.85%)	12 (26.09%)	10 (41.67%)	
Bachelor’s degree	33 (50.00%)	19 (41.30%)	10 (41.67%)	
Graduate degree	10(15.15%)	15 (32.61%)	4 (16.67%)	
Marital status	*n* (%)	*n* (%)	*n* (%)	0.871 ^b^
Married/partnered	37 (56.06%)	30 (65.22%)	13 (54.17%)	
Divorced/widowed	10(15.15%)	6 (13.04%)	4 (16.67%)	
Single, never partnered	19 (28.79%)	10 (21.74%)	7 (29.17%)	
Ethnicity	*n* (%)	*n* (%)	*n* (%)	0.719^b^
Black/African American	12(18.18%)	11 (23.91%)	4 (16.67%)	
White Non-Hispanic	43 (65.15%)	26 (56.52%)	13 (54.17%)	
Asian	6 (9.09%)	5 (10.87%)	2 (8.33%)	
Hispanic/Latino	4 (6.06%)	4 (8.70%)	4 (16.67%)	
Other	1 (1.52%)	0 (0.00%)	1 (4.17%)	
Employment	*n* (%)	*n* (%)	*n* (%)	0.206 ^b^
Unemployed	9 (13.64%)	7 (15.22%)	7 (29.17%)	
Employed	57 (86.36%)	39 (84.78%)	17 (70.83%)	
Surgery	*n* (%)	*n* (%)	*n* (%)	0.002 ^b^
Mastectomy	4 (6.06%)	7 (15.22%)	4 (16.67%)	
Lumpectomy	42 (63.64%)	12 (26.09%)	12 (50.00%)	
Breast reconstruction	20 (30.30%)	27 (58.70%)	8 (33.33%)	
Lymph nodes removed	Mean (SD)	Mean (SD)	Mean (SD)	
Nodes removed	2.72 (2.08)	4.28 (4.08)	3.54 (3.10)	0.0668 ^a^
	Median (IQR)	Median (IQR)	Median (IQR)	
Nodes removed	2.00 (1.00–3.00) A	4.00 (2.00–5.00) A	2.00 (2.00–5.00)	0.0368 ^c^
	*n* = 29	*n* = 31	*n* = 15	
Chemotherapy	*n* (%)	*n* (%)	*n* (%)	0.180^b^
Neoadjuvant	5 (17.24%)	11 (35.48%)	6 (40.00%)	
Adjuvant	24 (82.76%)	20 (64.52%)	9 (60.00%)	
Radiation	*n* (%)	*n* (%)	*n* (%)	0.652 ^b^
No	17 (28.33%)	15 (34.88%)	6 (25.00%)	
Yes	43 (71.67%)	28 (65.12%)	18 (75.00%)	
Cording at 8 weeks post-surgery	12(18.18%)	27 (62.79%)	16 (66.67%)	<0.001
Infections during the time of the study	12(18.18%)	4 (9.30%)	9 (37.50%)	0.018
Infections at 12 months post-surgery	6 (9.23%)	4 (8.7%)	9 (39.13%)	0.001
Physical therapy for limb mobility at 12 months post-surgery	18 (34.62%)	19 (61.29%)	14 (77.78%)	0.002
Moderate physical activity 2–3 times/week	32 (50.00%)	36 (78.26%)	12 (50.00%)	0.007

*A, B, C,* statistical pairwise differences between the three symptom risk classes with a Bonferroni correction for multiple comparisons at the 5% alpha level

a ANOVA

b Pearson Chi-square

c Kruskal-Wallis test

d SD, standard deviation

e IQR, interquartile range

**Table 5:** Demographic and clinical characteristics by symptom classes at 12 months post-surgery

Variables	Low symptom class Mean (SD)	Moderate symptom class Mean (SD)	Severe symptom class Mean (SD)	*p* value
Age	52.35 (11.13)	52.16 (10.73)	51.61 (12.49)	0.965 ^a^
Weight (pounds)	153.76 (35.70)	159.46 (33.23)	171.90 (39.94)	0.128^a^
BMI	26.41 (5.92)	28.02 (6.20)	29.90 (7.07)	0.079 ^a^
Education	n (%)	n (%)	n (%)	0.016^b^
Associates degree of less	11 (21.57%)	21 (33.87%)	13 (56.52%)	
Bachelor’s degree	24 (47.06%)	32 (51.61%)	6 (26.09%)	
Graduate degree	16(31.37%)	9 (14.52%)	4 (17.39%)	
Marital status	*n* (%)	*n* (%)	*n* (%)	0.960 ^b^
Married/partnered	31 (60.78%)	35 (56.45%)	14 (60.87%)	
Divorced/widowed	7 (13.73%)	9 (14.52%)	4 (17.39%)	
Single, never partnered	13 (25.49%)	18(29.03%)	5 (21.74%)	
Ethnicity	*n* (%)	*n* (%)	*n* (%)	0.187 ^b^
Black/African American	10(19.61%)	12 (19.35%)	5 (21.74%)	
White Non-Hispanic	29 (56.86%)	42 (67.74%)	11 (47.83%)	
Asian	5 (9.80%)	6 (9.68%)	2 (8.70%)	
Hispanic/Latino	5 (9.80%)	2 (3.23%)	5 (21.74%)	
Other	2 (3.92%)	0 (0.00%)	0 (0.00%)	
Employment	*n* (%)	*n* (%)	*n* (%)	0.448 ^b^
Unemployed	6 (11.76%)	12 (19.35%)	5 (21.74%)	
Employed	45 (88.24%)	50 (80.65%)	18 (78.26%)	
Surgery	*n* (%)	*n* (%)	*n* (%)	0.370 ^b^
Mastectomy	3 (5.88%)	7 (11.29%)	5 (21.74%)	
Lumpectomy	25 (49.02%)	31 (50.00%)	10 (43.48%)	
Breast reconstruction	23 (45.10%)	24 (38.71%)	8 (34.78%)	
	Mean (SD)	Mean (SD)	Mean (SD)	
Nodes removed	3.05 (2.41)	3.43 (3.52)	3.67 (2.50)	0.768 ^a^
	Median (IQR)	Median (IQR)	Median (IQR)	
Nodes removed	2.00 (1.00–4.50)	2.00 (2.00–4.00)	3.00 (2.00–5.00)	0.533 ^c^
	*n* = 22	*n* = 34	*n* = 19	
Chemotherapy	*n* (%)	*n* (%)	*n* (%)	0.021 ^b^
Neoadjuvant	3 (13.64%)	9 (26.47%)	10 (52.63%)	
Adjuvant	19 (86.36%)	25 (73.53%)	9 (47.37%)	
Radiation	*n* (%)	*n* (%)	*n* (%)	0.001 ^b^
No	23 (50.00%)	11 (18.97%)	4 (17.39%)	
Yes	23 (50.00%)	47 (81.03%)	19 (82.61%)	
Cording (axillary web syndrome) at 8 weeks post-surgery	16(32.00%)	24 (39.34%)	15 (68.18%)	0.015
Infections during the time of the study	6 (12.00%)	15 (24.59%)	4(18.18%)	0.239
Infection at 12 months post-surgery	3 (5.88%)	12 (19.67%)	4(18.18%)	0.096
Physical therapy for limb mobility at 12 months post-surgery	9 (24.32%)	26 (57.78%)	16 (84.21%)	
Moderate physical activity 2–3 times/week	29 (58.00%)	38 (62.30%)	13 (56.52%)	0.849

a ANOVA

b Pearson Chi-square

c Kruskal-Wallis test

d SD, standard deviation

e IQR, interquartile range

**Table 6:** Association between L-Dex and the symptom classes at 12 months post-surgery

Median (IQR) L-Dex	Low symptom class	Moderate symptom class	Severe symptom class	*p* value
Pre-surgery	− 0.60 (− 3.60–3.00)	0.35 (− 3.00–3.20)	− 0.50* (− 4.20–2.30)	0.687
8 weeks post-surgery	− 0.40 (− 3.60–1.80)	1.15 (− 2.20–5.70)	4.70* (0.60–7.80)	*0.002;* A < C
12 months post-surgery	− 1.00 (− 4.80–1.90)	0.15 (− 2.90–3.20)	4.40* (− 0.40–17.40)	< *0.001;* A, B < C

A (low), B (moderate), and C (severe) symptom class pairwise differences between the three classes with a Bonferroni correction at the 5% alpha level

* *p* < 0.05 - repeated measures ANOVA over the three time points

**Table 7:** Association between lymph volume change (LV%) and the symptom classes at 12 months post-surgery

Median (IQR) LV%	Low symptom class	Moderate symptom class	Severe symptom class	*p* value
Pre-surgery	− 1% (− 3–1%)	0% (− 2–2%)	− 1%*** (− 5–2%)	0.148
8 weeks post-surgery	− 1% (− 3–3%)	1% (− 2–4%)	3%*** (− 2–7%)	*0.041*
12 months post-surgery	− 1% (− 3–3%)	1% (− 2–5%)	6%*** (− 1–9%)	*0.001;* A < C

A (low), B (moderate), and C (severe) symptom class pairwise differences between the three classes with a Bonferroni correction at the 5% alpha level

* *p* < 0.05

** *p* <0.01

*** *p* < 0.001 – repeated measures ANOVA over the three time points

**Table 8:** Association between symptom distress subscales and the symptom classes at 12 months post-surgery

Median (IQR) symptom distress	Low symptom class	Moderate symptom class	Severe symptom class	*p* value
Impaired daily living	0.00 (0.00–0.00)	0.00 (0.00–2.00)	8.00 (2.00–13.00)	*0.0001;* A<B<C
Social distress	0.00 (0.00–0.00)	0.00 (0.00–0.00)	1.00 (0.00–3.00)	*0.0001;* A, B<C
Emotional distress	0.00 (0.00–0.00	1.00 (0.00–4.00)	11.00 (4.00–12.00)	*0.0001;* A<B<C
Impaired self-perception	0.00 (0.00–0.00)	1.00 (0.00–1.00)	2.00 (1.00–2.00)	*0.0001;* A<B<C
Sleep disturbance	0.00 (0.00–0.00)	0.00 (0.00–1.00)	2.00 (0.00–3.00)	*0.0001;* A<B<C
Impaired sexuality	0.00 (0.00–0.00)	0.00 (0.00–0.00)	1.00 (0.00–2.00)	*0.0001;* A, B<C
Work outside home	0.00 (0.00–0.00)	0.00 (0.00–1.00)	1.50 (0.00–2.00)	*0.0001;* A<B<C
Days absent from work	0.00 (0.00–0.00)	0.00 (0.00–16.00)	3.50 (0.00–21.00)	*0.0001;* A<B, C

A (low), B (moderate), and C (severe) symptom class pairwise differences between the three classes with a Bonferroni correction at the 5% alpha level

**Table 9:** Associations between biomarkers and lymphedema symptom classes at 8 weeks post-surgery

Biomarkers pg/mL	Low symptom class	Moderate symptom class	Severe symptom class	*p* value
Mean Ln(IL1-*α*) levels				
Pre-surgery	0.95 (1.76)	− 0.25 (1.62)	− 1.92 (1.69)	0.0012; A>C
8 weeks post-surgery	0.20 (1.45)	− 0.46 (1.35)	− 1.90 (1.63)	0.0079; A>C
12 months post-surgery	− 1.01 (1.86)	− 0.97 (0.90)	− 1.94 (0.79)	0.5432
Mean Ln(IL-4) levels				
Pre-surgery	− 3.48 (0.92)	− 3.33 (1.01)	− 2.83 (1.31)	0.0525
8 weeks post-surgery	− 3.46 (1.01)	− 3.42(1.08)	− 2.85 (1.21)	0.0747
12 months post-surgery	− 3.31 (1.41)	− 3.63 (0.96)	− 2.99 (1.54)	0.2226
Mean Ln(IL-6) levels				
Pre-surgery	0.12* (1.32)	0.28* (1.52)	1.15 (1.79)	0.0168; A<C
4–8 weeks post-surgery	0.38* (1.34)	0.66* (1.51)	1.17 (1.67)	0.0835
12 months post-surgery	0.30* (1.46)	0.45* (1.68)	0.78 (1.94)	0.4838
Mean Ln(IL-8) levels				
Pre-surgery	2.39 (0.99)	2.11 (1.08)	1.63 (1.78)	0.0334; A>C
4–8 weeks post-surgery	2.36(1.15)	2.04 (1.18)	1.98 (1.79)	0.3358
12 months post-surgery	2.45 (1.04)	2.18 (1.23)	2.09 (1.19)	0.3171
Mean Ln(IL-10) levels				
Pre-surgery	− 1.38 (0.66)	−1.49 (0.61)	− 1.61 (1.39)	0.5250
4–8 weeks post-surgery	− 1.40 (0.80)	−1.56 (0.84)	− 1.60 (1.39)	0.5966
12 months post-surgery	− 1.25 (0.94)	−1.50 (0.51)	− 1.01 (1.06)	0.1060
Mean Ln(IL-13) levels				
Pre-surgery	− 1.07 (1.19)	−1.05 (1.16)	− 1.75 (1.33)	0.2814
4–8 weeks post-surgery	1.46 (1.34)	−1.95 (2.11)	− 2.21 (1.88)	0.4162
12 months post-surgery	− 1.04 (1.58)	− 0.67 (1.79)	− 1.41 (1.72)	0.4407
Mean Ln(VEGF) levels				
Pre-surgery	4.54** (0.62)	4.75* (0.78)	4.90*** (0.71)	0.0681
8 weeks post-surgery	4.50** (0.65)	4.73* (0.78)	4.96*** (0.80)	0.0290; A< C
12 months post-surgery	4.27** (0.83)	4.56* (0.78)	4.59*** (0.81)	0.1130
Mean Ln(VEGFC) levels				
Pre-surgery	6.09** (0.44)	6.03* (0.56)	6.12 (0.47)	0.6978
4–8 weeks post-surgery	5.88** (0.50)	5.89* (0.67)	6.04 (0.49)	0.4847
12 months post-surgery	5.93** (0.52)	5.97* (0.37)	5.92 (0.53)	0.8746
Mean Ln(VEGFD) levels				
Pre-surgery	7.34*** (0.62)	7.26*** (0.54)	7.30* (0.48)	0.7835
4–8 weeks post-surgery	6.88*** (0.44)	6.76*** (0.40)	6.98* (0.51)	0.1380
12 months post-surgery	7.03*** (0.63)	6.95*** (0.54)	7.18* (0.53)	0.3228
Mean Ln(TNF-*α*) levels				
Pre-surgery	0.65*** (0.40)	0.55*** (0.40)	0.49 (1.20)	0.5697
4–8 weeks post-surgery	0.81*** (0.56)	0.85*** (0.35)	0.73 (0.96)	0.7381
12 months post-surgery	0.79*** (0.44)	0.75*** (0.38)	0.67 (0.43)	0.5138

A (low), B (moderate), and C (severe) symptom class pairwise differences between the three classes with a Bonferroni correction at the 5% alpha level

* *p* < 0.05

** *p* <0.01

*** *p* < 0.001 – repeated measures ANOVA over the three time points show changes over time

**Table 10:** Associations between biomarkers and lymphedema symptom classes at 12 months post-surgery

Biomarkers pg/mL	Low symptom class	Moderate symptom class	Severe symptom class	*p* value
Mean Ln(IL1-a) levels				
Pre-surgery	− 0.03* (1.20)	0.00 (2.63)	− 0.46 (1.93)	0.8837
4–8 weeks post-surgery	− 0.09* (1.38)	− 0.19(1.39)	− 1.27 (2.12)	0.1692
12 months post-surgery	− 1.41* (1.41)	− 0.85 (1.64)	− 1.27 (1.96)	0.6480
Mean Ln(IL-4) levels				
Pre-surgery	− 3.05 (1.10)	− 3.49 (0.99)	− 3.34 (1.06)	0.1350
4–8 weeks post-surgery	− 3.03 (1.10)	− 3.55 (1.01)	− 3.31 (1.20)	0.1011
12 months post-surgery	− 3.05 (1.23)	− 3.53 (1.41)	− 3.33 (1.23)	0.2763
Mean Ln(IL-6) levels				
Pre-surgery	0.64 (1.78)	0.15** (1.29)	0.43 (1.50)	0.2570
4–8 weeks post-surgery	0.89 (1.72)	0.39** (1.23)	0.73 (1.58)	0.2183
12 months post-surgery	0.64 (2.00)	0.25** (1.41)	0.56 (1.35)	0.4664
Mean Ln(IL-8) levels				
Pre-surgery	2.09 (1.27)	2.22 (1.22)	2.08* (1.20)	0.8216
4–8 weeks post-surgery	1.98 (1.60)	2.25 (1.16)	2.36* (0.95)	0.4278
12 months post-surgery	2.35 (1.42)	2.32 (0.99)	2.08* (0.87)	0.6175
Mean Ln(IL-10) levels				
Pre-surgery	− 1.69 (0.73)	−1.30 (0.65)	− 1.43 (1.28)	0.0699
4–8 weeks post-surgery	− 1.61 (0.94)	−1.43 (0.84)	− 1.41 (1.17)	0.6101
12 months post-surgery	− 1.34 (0.90)	−1.26 (0.93)	− 1.33 (0.42)	0.9007
Mean Ln(IL13) levels				
Pre-surgery	− 1.26 (1.20)	−1.14 (1.28)	− 1.22 (1.27)	0.9505
4–8 weeks post-surgery	− 2.03 (2.05)	−1.66 (1.49)	− 1.55 (1.59)	0.6979
12 months post-surgery	− 1.01 (1.96)	− 0.86(1.51)	− 1.32 (1.34)	0.7619
Mean Ln(VEGF) levels				
Pre-surgery	4.53 (0.70)	4.67** (0.69)	4.98** (0.70)	0.0477; A<C
4–8 weeks post-surgery	4.52 (0.73)	4.66** (0.70)	4.98** (0.82)	0.0571
12 months post-surgery	4.37 (0.62)	4.42** (0.81)	4.61** (1.14)	0.5169
Mean Ln(VEGFC) levels				
Pre-surgery	6.15* (0.40)	6.09** (0.44)	5.88 (0.70)	0.0938
4–8 weeks post-surgery	5.98* (0.48)	5.90** (0.47)	5.78 (0.86)	0.3732
12 months post-surgery	5.99* (0.38)	5.94** (0.50)	5.85 (0.59)	0.5346
Mean Ln(VEGFD) levels				
Pre-surgery	7.39*** (0.60)	7.33*** (0.56)	7.05 (0.42)	0.0571
4–8 weeks post-surgery	6.90*** (0.45)	6.83*** (0.47)	6.84 (0.38)	0.7112
12 months post-surgery	7.10*** (0.63)	7.02*** (0.55)	6.93 (0.56)	0.5118
Mean Ln(TNF-*α*) levels				
Pre-surgery	0.60** (0.64)	0.56*** (0.41)	0.59 (1.05)	0.9502
4–8 weeks post-surgery	0.81** (0.70)	0.84*** (0.42)	0.73 (0.82)	0.7779
12 months post-surgery	0.73** (0.49)	0.76*** (0.39)	0.77 (0.36)	0.9266

A (low), B (moderate), and C (severe) symptom class pairwise differences between the three classes with a Bonferroni correction at the 5% alpha level

* *p* < 0.05

** *p* <0.01

*** *p* < 0.001 – repeated measures ANOVA over the three time points show changes over time

**Table 11:** Biomarker Levels and Lymphedema Symptom Classes at 8 Weeks Post-surgery

Biomarkers pg/mL	Low symptom class	Moderate symptom class	Severe symptom class
	Median IL1-*α* levels (IQR)		
Pre-surgery	0.00 (0.00–0.42)	0.00 (0.00–0.60)	0.00 (0.00–0.06)
8 weeks post-surgery	0.00 (0.00–0.40)	0.00 (0.00–0.28)	0.00 (0.00–0.04)
12 months post-surgery	0.00 (0.00–0.08)	0.00 (0.00–0.00)	0.00 (0.00–0.00)
	Median IL-4 levels (IQR)		
Pre-surgery	0.03 (0.01–0.04)	0.03 (0.01–0.05)	0.03 (0.02–0.15)
8 weeks post-surgery	0.02 (0.01–0.04)	0.02 (0.01–0.03)	0.04 (0.02–0.11)
12 months post-surgery	0.02 (0.01–0.04)	0.02 (0.00–0.03)	0.03 (0.02–0.13)
	Median IL-6 levels (IQR)		
Pre-surgery	0.75 (0.47–1.59)	0.80 (0.48–2.18)	1.42 (0.81–33.03)
4–8 weeks post-surgery	0.98 (0.62–2.06)	1.15 (0.73–2.27)	2.04 (1.03–24.77)
12 months post-surgery	0.90 (0.55–1.98)	1.01 (0.67–2.03)	1.09 (0.67–12.76)
	Median IL-8 levels (IQR)		
Pre-surgery	13.53 (10.34–16.29)	10.55 (7.39–14.83)	11.62 (0.76–15.21)
4–8 weeks post-surgery	14.36 (9.87–17.57)	11.89 (7.77–14.88)	15.35 (1.10–21.53)
12 months post-surgery	12.79 (9.58–18.34)	10.59 (7.33–16.41)	12.27 (5.42–16.07)
	Median IL-10 levels (IQR)		
Pre-surgery	0.24 (0.16–0.33)	0.21 (0.16–0.33)	0.26 (0.09–0.36)
4–8 weeks post-surgery	0.24 (0.19–0.38)	0.24 (0.14–0.37)	0.30 (0.03–0.40)
12 months post-surgery	0.27 (0.18–0.40)	0.21 (0.17–0.32)	0.29 (0.10–0.35)
	Median IL-13 levels (IQR)		
Pre-surgery	0.00 (0.00–0.32)	0.00 (0.00–0.39)	0.00 (0.00–0.08)
4–8 weeks post-surgery	0.00 (0.00–0.24)	0.00 (0.00–0.24)	0.01 (0.00–0.11)
12 months post-surgery	0.00 (0.00–0.35)	0.03 (0.00–0.54)	0.04 (0.00–0.40)
	Median VEGF levels (IQR)		
Pre-surgery	96.21 (54.66–145.58)	132.43 (69.98–215.01)	139.86 (97.77–198.80)
8 weeks post-surgery	99.53 (57.64–138.15)	123.44 (71.73–209.47)	155.61 (89.28–256.05)
12 months post-surgery	78.95 (40.12–127.17)	102.82 (56.60–163.36)	84.89 (63.75–215.97)
	Median VEGFC levels (IQR)		
Pre-surgery	466.17 (335.20–603.83)	448.37 (299.87–562.69)	474.30 (335.16–642.67)
4–8 weeks post-surgery	364.13 (263.94–515.48)	381.76 (276.47–541.35)	400.86 (323.74–589.70)
12 months post-surgery	387.00 (276.10–500.60)	402.10 (312.04–474.40)	374.18 (259.90–536.97)
	Median VEGFD levels (IQR)		
Pre-surgery	1661.01 (932.11–2440.33)	1390.60 (969.25–2048.68)	1439.30 (1072.33–2205.00)
4–8 weeks post-surgery	964.49 (730.50–1413.14)	898.66 (654.56–1137.88)	1012.76 (765.34–1356.22)
12 months post-surgery	1011.67 (746.80–1526.07)	1045.40 (709.52–1472.45)	1374.00 (832.79–1772.05)
	Median TNF-*α* levels (IQR)		
Pre-surgery	1.88 (1.37–2.40)	1.90 (1.23–2.31)	1.86 (1.32–2.48)
4–8 weeks post-surgery	2.43 (1.88–2.99)	2.16 (1.60–3.19)	2.63 (1.90–2.84)
12 months post-surgery	2.14 (1.79–2.86)	2.12 (1.63–2.59)	2.24 (1.34–2.48)

**Table 12:** Biomarker levels and lymphedema symptom classes at 12 months post-surgery

Biomarkers pg/mL	Low symptom class	Moderate symptom class	Severe symptom class
	Median IL1-*α* levels (IQR)		
Pre-surgery	0.00 (0.00–0.73)	0.00 (0.00–0.07)	0.00 (0.00–0.06)
8 weeks post-surgery	0.00 (0.00–0.41)	0.00 (0.00–0.04)	0.00 (0.00–0.09)
12 months post-surgery	0.00 (0.00–0.01)	0.00 (0.00–0.03)	0.00 (0.00–0.00)
	Median IL-4 levels (IQR)		
Pre-surgery	0.03 (0.02–0.06)	0.02 (0.01–0.04)	0.03 (0.02–0.05)
8 weeks post-surgery	0.02 (0.02–0.07)	0.02 (0.01–0.03)	0.03 (0.01–0.04)
12 months post-surgery	0.03 (0.00–0.04)	0.02 (0.01–0.03)	0.02 (0.02–0.04)
	Median IL-6 levels (IQR)		
Pre-surgery	1.01 (0.53–4.15)	0.81 (0.48–1.76)	0.94 (0.53–2.88)
4–8 weeks post-surgery	1.09 (0.63–18.32)	1.20 (0.68–1.92)	1.11 (0.68–4.36)
12 months post-surgery	1.02 (0.58–4.04)	0.90 (0.59–1.67)	1.07 (0.75–2.12)
	Median IL-8 levels (IQR)		
Pre-surgery	12.90 (9.29–16.07)	12.45 (9.35–16.05)	11.34 (7.05–15.36)
4–8 weeks post-surgery	13.39 (6.88–17.16)	13.26 (8.65–16.60)	14.24 (10.06–17.29)
12 months post-surgery	13.26 (9.81–18.95)	12.10 (9.11–17.40)	10.49 (8.15–12.90)
	Median IL-10 levels (IQR)		
Pre-surgery	0.18 (0.13–0.31)	0.24 (0.18–0.33)	0.28 (0.14–0.44)
4–8 weeks post-surgery	0.22 (0.15–0.35)	0.27 (0.19–0.39)	0.25 (0.19–0.42)
12 months post-surgery	0.25 (0.15–0.34)	0.25 (0.18–0.36)	0.24 (0.18–0.33)
	Median IL-13 levels (IQR)		
Pre-surgery	0.00 (0.00–0.37)	0.00 (0.00–0.20)	0.00 (0.00–0.10)
4–8 weeks post-surgery	0.00 (0.00–0.27)	0.00 (0.00–0.16)	0.00 (0.00–0.24)
12 months post-surgery	0.05 (0.00–0.41)	0.00 (0.00–0.37)	0.00 (0.00–0.34)
	Median VEGF levels (IQR)		
Pre-surgery	85.38 (60.67–138.82)	121.94 (62.20–166.97)	162.17 (103.54–204.53)
8 weeks post-surgery	99.34 (57.17–145.09)	109.35 (74.77–169.76)	171.58 (107.19–267.32)
12 months post-surgery	80.80 (56.03–126.35)	87.02 (51.35–153.98)	148.21 (66.09–228.92)
	Median VEGFC levels (IQR)		
Pre-surgery	491.87 (380.78–603.66)	455.74 (309.15–601.06)	423.56 (274.88–527.12)
4–8 weeks post-surgery	381.76 (292.92–567.95)	368.43 (264.49–536.84)	342.16 (236.43–543.47)
12 months post-surgery	418.44 (312.04–506.92)	388.51 (283.97–526.38)	373.88 (218.23–468.71)
	Median VEGFD levels (IQR)		
Pre-surgery	1698.92 (1082.71–2573.46)	1696.59 (966.92–2302.68)	1090.51 (801.47–1448.77)
4–8 weeks post-surgery	987.30 (705.97–1434.41)	905.38 (730.66–1232.77)	985.37 (741.19–1230.39)
12 months post-surgery	1251.46 (804.47–1633.44)	1082.99 (771.10–1720.71)	894.15 (706.84–1174.30)
	Median TNF-*α* levels (IQR)		
Pre-surgery	1.82 (1.31–2.15)	1.91 (1.24–2.34)	2.11 (1.46–2.90)
4–8 weeks post-surgery	2.21 (1.49–2.73)	2.44 (1.92–2.99)	2.55 (1.69–3.19)
12 months post-surgery	2.10 (1.32–2.65)	2.15 (1.79–2.81)	2.39 (1.86–2.71)

## Highlights

- Multiple lymphedema symptoms occur concurrently following breast cancer surgery.
- Latent class analysis can distinguish low, moderate, and severe lymphedema symptom classes.
- The severe symptom class had the highest symptom distress and highest limb volume and lymph fluid level.
- The moderate symptom class may indicate an early stage of arm lymphedema.
- Biomarkers may be used to predict risk for lymphedema symptoms.

## References

[1] Breast cancer facts & figures 2019–2020. Atlanta: American Cancer Society (ACS); 2020.

[2] Paskett ED, Naughton MJ, McCoy TP, Case LD, Abbott JM. The epidemiology of arm and hand swelling in premenopausal breast cancer survivors. Cancer Epidemiol Biomarks Prev. 2007;16(4): 775–82. 10.1158/1055-9965.EPI-06-0168 PMC4771019 17416770

[3.•] Armer JM, Stewart BR. A comparison of four diagnostic criteria for lymphedema in a post-breast cancer population. Lymphat Res Biol. 2005;3(4):208–17. 16379589 10.1089/lrb.2005.3.208

[4] Fu MR, Conley YP, Axelrod D, Guth AA, Yu G, Fletcher J, Precision assessment of heterogeneity of lymphedema phenotype, genotypes and risk prediction. Breast Edinb Scotl. 2016;29:231–40. 10.1016/j.breast.2016.06.023 PMC5014618 27460425

[5] Petrek JA, Senie RT, Peters M, Rosen PP. Lymphedema in a cohort of breast carcinoma survivors 20 years after diagnosis. Cancer. 2001;92(6):1368–77. 11745212 10.1002/1097-0142(20010915)92:6<1368::aid-cncr1459>3.0.co;2-9

[6] Armer JM, Radina ME, Porock D, Culbertson SD. Predicting breast cancer-related lymphedema using self-reported symptoms. Nurs Res. 2003;52(6):370–9. 14639083 10.1097/00006199-200311000-00004

[7] Fu MR, Rosedale M. Breast cancer survivors’ experiences of lymphedema-related symptoms. J Pain Symptom Manag. 2009;38(6):849–59. 10.1016/j.jpainsymman.2009.04.030 PMC2795115 19819668

[8] Fu MR, Axelrod D, Cleland CM, Qiu Z, Guth AA, Kleinman R, Symptom report in detecting breast cancer-related lymphedema. Breast Cancer Dove Med Press. 2015;7:345–52. 26527899 10.2147/BCTT.S87854 PMC4621182

[9] Fu MR, Deng J, Armer JM. Putting evidence into practice: cancer-related lymphedema. Clin J Oncol Nurs. 2014;18(Suppl):68–79. 25427610 10.1188/14.CJON.S3.68-79

[10] Deng J, Fu MR, Armer JM, Cormier JN, Radina ME, Thiadens SRJ, Factors associated with reported infection and lymphedema symptoms among individuals with extremity lymphedema. Rehabil Nurs. 2015;40(5):310–9. 25042377 10.1002/rnj.171

[11] Mak SS, Yeo W, Lee YM, Mo KF, Tse KY, Tse SM, Predictors of lymphedema in patients with breast cancer undergoing axillary lymph node dissection in Hong Kong. Nurs Res. 2008;57(6):416–25. 19018216 10.1097/NNR.0b013e31818c3de2

[12] Newman B, Lose F, Kedda M-A, Francois M, Ferguson K, Janda M, Possible genetic predisposition to lymphedema after breast cancer. Lymphat Res Biol. 2012;10(1):2–13. 22404826 10.1089/lrb.2011.0024 PMC3311400

[13] Leung G, Baggott C, West C, Elboim C, Paul SM, Cooper BA, Cytokine candidate genes predict the development of secondary lymphedema following breast cancer surgery. Lymphat Res Biol. 2014;12(1):10–22. 24502445 10.1089/lrb.2013.0024 PMC3961780

[14] Smoot B, Kober KM, Paul SM, Levine JD, Abrams G, Mastick J, Potassium channel candidate genes predict the development of secondary lymphedema following breast cancer surgery. Nurs Res. 2017;66(2):85–94. 28252570 10.1097/NNR.0000000000000203 PMC5334662

[15] Miaskowski C, Dodd M, Paul SM, West C, Hamolsky D, Abrams G, Lymphatic and angiogenic candidate genes predict the development of secondary lymphedema following breast cancer surgery. Lafrenie R, editor. PLoS One. 2013;8(4):e60164. 23613720 10.1371/journal.pone.0060164 PMC3629060

[16] Fu MR, Cleland CM, Guth AA, Kayal M, Haber J, Cartwright F, L-dex ratio in detecting breast cancer-related lymphedema: reliability, sensitivity, and specificity. Lymphology. 2013;46(2): 85–96. 24354107 PMC4040962

[17] Fu MR, Axelrod D, Guth AA, Cartwright F, Qiu Z, Goldberg JD, Proactive approach to lymphedema risk reduction: a prospective study. Ann Surg Oncol. 2014;21(11):3481–9. 24809302 10.1245/s10434-014-3761-z PMC4163073

[18] Fu MR, Axelrod D, Guth AA, Rampertaap K, El-Shammaa N, Hiotis K, mHealth self-care interventions: managing symptoms following breast cancer treatment. mHealth. 2016 7;2. 10.21037/mhealth.2016.07.03 PMC4970761 27493951

[19] Shi S, Lu Q, Fu MR, Ouyang Q, Liu C, Lv J, Psychometric properties of the breast cancer and lymphedema symptom experience index: the Chinese version. Eur J Oncol Nurs. 2016;20:10–6. 26071198 10.1016/j.ejon.2015.05.002

[20] Measuring physical activity intensity | physical activity | CDC [Internet]. Centers for Disease Control and Prevention (CDC). 2020 [cited 2020 May 9]. Available from: https://www.cdc.gov/physicalactivity/basics/measuring/index.html

[21] Rindskopf D, Rindskopf W. The value of latent class analysis in medical diagnosis. Stat Med. 1986 1;5(1):21–7. 3961312 10.1002/sim.4780050105

[22] Cheung YK, Yu G, Wall MM, Sacco RL, Elkind MSV, Willey JZ. Patterns of leisure-time physical activity using multivariate finite mixture modeling and cardiovascular risk factors in the Northern Manhattan Study. Ann Epidemiol. 2015;25(7):469–74. 25873383 10.1016/j.annepidem.2015.03.003 PMC4457695

[23] Yu G, Wall MM, Chiasson MA, Hirshfield S. Complex drug use patterns and associated HIV transmission risk behaviors in an Internet sample of U.S. men who have sex with men. Arch Sex Behav. 2015;44(2):421–8. 25104104 10.1007/s10508-014-0337-8 PMC4381804

[24] Fraley C How many clusters? Which clustering method? Answers via model-based cluster analysis. Comput J. 1998;41(8):578–88.

[25] McLachlan GJ, Peel D. Finite mixture models. Wiley; 2004. 450 p.

[26] Muthén L, Muthén B. Mplus. Statistical analysis with latent variables. User’s Guide. 2006.

[27] Dinarello CA, Pomerantz BJ. Proinflammatory cytokines in heart disease. Blood Purif. 2001;19(3):314–21. 11244192 10.1159/000046960

[28] Laban-Guceva N, Bogoev M, Antova M. Serum concentrations of interleukin (IL-)1alpha, 1beta, 6 and tumor necrosis factor (TNF-) alpha in patients with thyroid eye disease (TED). Med Arh. 2007;61(4):203–6. 18297990

[29] Biancotto A, Wank A, Perl S, Cook W, Olnes MJ, Dagur PK, Baseline levels and temporal stability of 27 multiplexed serum cytokine concentrations in healthy subjects. Boasso A, editor. PLoS One. 2013;8(12):e76091. 24348989 10.1371/journal.pone.0076091 PMC3861126

[30] Linderholm BK, Hellborg H, Johansson U, Elmberger G, Skoog L, Lehtiö J, Significantly higher levels of vascular endothelial growth factor (VEGF) and shorter survival times for patients with primary operable triple-negative breast cancer. Ann Oncol. 2009;20(10):1639–46. 19549711 10.1093/annonc/mdp062

